# *Hydnophanerochaete* and *Odontoefibula*, two new genera of phanerochaetoid fungi (Polyporales, Basidiomycota) from East Asia

**DOI:** 10.3897/mycokeys.39.28010

**Published:** 2018-09-17

**Authors:** Che-Chih Chen, Sheng-Hua Wu, Chi-Yu Chen

**Affiliations:** 1 Department of Plant Pathology, National Chung Hsing University, Taichung 40227, Taiwan National Chung Hsing University Taichung Taiwan; 2 Department of Biology, National Museum of Natural Science, Taichung 40419, Taiwan National Museum of Natural Science Taichung Taiwan

**Keywords:** Meruliaceae, multi-marker phylogeny, new species, Phanerochaetaceae, phlebioid clade

## Abstract

Two new genera with phylogenetic affinities to *Phanerochaete* s.l. are presented, namely *Hydnophanerochaete* and *Odontoefibula*. The generic type of *Hydnophanerochaete* is *Phanerochaeteodontoidea*. *Odontoefibula* is established based on a new species: *O.orientalis* (generic type). Both genera have effused basidiocarps with odontioid hymenial surface, simple-septate generative hyphae, cystidia lacking, clavate basidia and ellipsoid basidiospores that are smooth, thin-walled, inamyloid, non-dextrinoid and acyanophilous. *Hydnophanerochaete* is additionally characterised by a compact texture in the subiculum with thick-walled generative hyphae and quasi-binding hyphae. *Odontoefibula* has a dense texture of subiculum with thin- to slightly thick-walled hyphae and further a dark reddish reaction of basidiocarps when treated with KOH. Multi-marker phylogenetic analyses based on sequences, inferred from the ITS+nuc 28S+*rpb1*+*rpb2*+*tef1* dataset, indicate that *Hydnophanerochaete* and *Odontoefibula* are placed in the Meruliaceae and *Donkia* clades of Phanerochaetaceae, respectively. *Phanerochaetesubodontoidea* is a synonym of *P.odontoidea*, according to morphological and molecular evidence.

## Introduction

The genus *Phanerochaete* P. Karst., typified by *P.alnea* (Fr.) P. Karst., belongs to Polyporales Gäum of the Basidiomycota R.T. Moore and is one of the largest genera of corticoid fungi, including over 150 names according to Index Fungorum (http://www.indexfungorum.org/). Basidiocarps are typically membranaceous, effused, with various hymenial surfaces (i.e. smooth, tuberculate, odontioid, hydnoid, merulioid or poroid). Microscopically, *Phanerochaete* has a monomitic hyphal system, ordinarily simple-septate generative hyphae (rare clamp connections can be found in the subiculum), ellipsoid to cylindrical thin-walled basidiospores and clavate basidia. *Phanerochaete* is widespread and grows on diverse woody substrates (i.e. twigs and branches or trunks of angiosperms or gymnosperms), causing a white rot. *Phanerochaete* s.l. has attracted increasing study interest due to its abundant taxonomic diversity and potential applications in the field of biodegradation and bioconversion (Sánchez 2009).

*Phanerochaete* was traditionally treated as a genus in the broad sense (Eriksson et al. 1978; Burdsall 1985; Wu 1990). In recent years, *Phanerochaete* has been shown to be a polyphyletic group with members distributed throughout the phlebioid clade of Polyporales (De Koker et al. 2003; Wu et al. 2010; Floudas and Hibbett 2015; Miettinen et al. 2016), which was recently recognised as three families: Phanerochaetaceae Jülich, Irpicaceae Spirin & Zmitr and Meruliaceae Rea (Justo et al. 2017). Based on the combined morphological and molecular approaches, many studies have been conducted to revise the generic concept of *Phanerochaete* s.l. Some segregated genera have been recovered or proposed, e.g. *Efibula* Sheng H. Wu, *Hydnophlebia* Parmasto, *Phaeophlebiopsis* Floudas & Hibbett, *Phlebiopsis* Jülich, *Rhizochaete* Gresl., Nakasone & Rajchenb. and *Scopuloides* (Massee) Höhn. & Litsch. (Wu 1990; Greslebin et al. 2004; Wu et al. 2010; Floudas and Hibbett 2015).

*Phanerochaeteodontoidea* Sheng H. Wu and *P.subodontoidea* Sheng H. Wu were described from Taiwan (Wu 2000). Both species have ceraceous basidiocarps with odontioid to hydnoid hymenial surface, compact subiculum, but no cystidia. These species have been shown to be phylogenetically far from the core *Phanerochaete* clade (Wu et al. 2010; Ghobad-Nejhad et al. 2015; Wu et al. 2018) and were placed by Justo et al. (2017) in Meruliaceae. In this study, we evaluate the generic placement of *P.odontoidea* and *P.subodontoidea*, as well as morphologically similar species. To accommodate our target taxa, we found it necessary to introduce two new genera placed within Meruliaceae and Phanerochaetaceae, respectively.

When *Phanerochaeteodontoidea* and *P.subodontoidea* were described, they were separated by basidiospore width (Wu 2000). After 2000, we have accumulated more collections identified as *P.odontoidea* and *P.subodontoidea* from China, Japan, Taiwan and Vietnam. To better reflect their morphological variations, this study provides updated morphological and molecular evidence for revising their species concepts.

## Materials and methods

### *Morphological studies*

The specimens used for illustrations and descriptions are deposited at the herbarium of National Museum of Natural Science of ROC (TNM, acronym according to Index Herbariorum; http://sweetgum.nybg.org/science/ih/). Free-hand thin sections of basidiocarps were mounted in three mounting media for microscopic studies: 5% (w/v) KOH with 1% (w/v) phloxine was used for observation and measurements; Melzer’s reagent (IKI) was utilised to check amyloidity and dextrinoidity; and Cotton Blue (CB, Fluka 61335) was employed to determine cyanophily. Sections were studied with a Leica DM2500 (Leica, Wetzlar) microscope. Drawings were done with the aid of a drawing tube. We followed the method for measurements of microscopic characters by Wu (1990). The abbreviations below were used when presenting statistical measurements of basidiospores: L = mean basidiospore length, W = mean basidiospore width, Q = variation in L/W ratio, n = number of measured spores. The terminology of microscopic characters follows Wu (1990).

### *DNA extraction and sequencing*

Dried specimens or mycelia grown on MEA were used for isolating genomic DNA. The material was first fragmented into a fine powder with the aid of liquid nitrogen and a TissueLyser II (Qiagen, Hilden, Germany). DNA was obtained using the Plant Genomic DNA Extraction Miniprep System (Viogene-Biotek Corp., New Taipei, Taiwan) based on the manufacturer’s instructions. Five genetic markers were amplified in this study: nuc rDNA ITS1-5.8S-ITS2 (ITS) using primer pair ITS1/ITS4 (White et al. 1990); D1-D2 domains of nuc 28S rDNA (nuc 28S) using primer pair LR0R/LR5 (http://www2.clarku.edu/faculty/dhibbett/Protocols_Folder/Primers/Primers.pdf); RNA polymerase II largest subunit (*rpb1*) using primer pair RPB1-Af/RPB1-Cr (Stiller and Hall 1997; Matheny et al. 2002) or alternative primers RPB1- 2f, RPB1-2.1f, RPB1-2.2f and RPB1-2.1r (Frøslev et al. 2005); RNA polymerase II second largest subunit (*rpb2*) using primer pair RPB2-f5F/RPB2-b7.1R (Liu et al. 1999; Matheny 2005); and translation elongation factor 1-α (*tef1*) using primer pair EF1-983F/EF1-2212R (Rehner and Buckley 2005). The PCR protocols for ITS and nuc 28S gene regions were as follows: initial denaturation at 95 °C for 5 min, followed by 40 cycles at 94 °C for 45 s, 53 °C for ITS and 50 °C for nuc 28S for 45 s and 72 °C for 45 s and a final extension of 72 °C for 10 min. The PCR protocols for *rpb1*, *rpb2* and *tef1* include initial denaturation at 94 °C for 2 min, followed by 35 cycles at 94 °C for 40 s, 60 °C for 40 s and 72 °C for 2 min and a final extension of 72 °C for 10 min. PCR products were purified and sequenced by the MB Mission Biotech Company (Taipei, Taiwan). Newly obtained sequences for each of the five markers were assembled and manually adjusted using BioEdit (Hall 1999) and then submitted to the DNA Data Bank of Japan (DDBJ) (http://www.ddbj.nig.ac.jp/; Table 1). We have verified the accuracy and identity of consensus sequences by comparing with sequences in GenBank (https://www.ncbi.nlm.nih.gov/genbank/).

**Table 1.** Species and sequences used in the phylogenetic analyses. Newly generated sequences are set in bold.

Taxon	Strain/Specimen	ITS	nuc 28S	*rpb1*	*rpb2*	*tef1*
* Antrodia serialis *	KHL 12010 (GB)	JX109844	JX109844	–	JX109870	JX109898
* Aurantiporus croceus *	Miettinen-16483	KY948745	KY948901	KY948927	–	–
* Bjerkandera adusta *	HHB-12826-Sp	KP134983	KP135198	KP134784	KP134913	KT305938
Bjerkandera aff. centroamericana	L-13104-sp	KY948791	KY948855	KY948936	–	–
* Byssomerulius corium *	FP-102382	KP135007	KP135230	KP134802	KP134921	–
* Candelabrochaete africana *	FP-102987-Sp	KP135294	KP135199	KP134872	KP134975	–
* Ceraceomyces serpens *	HHB-15692-Sp	KP135031	KP135200	KP134785	KP134914	–
* Ceriporia alachuana *	FP-103881-Sp	KP135341	KP135201	KP134845	KP134896	–
* Ceriporia reticulata *	KHL 11981 (GB)	–	–	–	–	JX109899
* Ceriporia reticulata *	RLG-11354-Sp	KP135041	KP135204	KP134794	KP134922	–
* Ceriporiopsis aneirina *	HHB-15629-Sp	KP135023	KP135207	KP134795	–	–
* Ceriporiopsis carnegieae *	RLG-7277-T	KY948792	KY948854	KY948935	–	–
* Ceriporiopsis fimbriata *	Dai 11672	KJ698633	KJ698637	–	–	–
* Ceriporiopsis gilvescens *	L-3519-sp	KY948761	–	KY948919	–	–
* Ceriporiopsis gilvescens *	Niemela-5516	–	HQ659222	–	–	–
* Ceriporiopsis guidella *	HUBO 7659	FJ496687	FJ496722	–	–	–
* Ceriporiopsis kunmingensis *	C.L. Zhao 152	KX081072	KX081074	–	–	–
* Ceriporiopsis lagerheimii *	58240	KX008365	KX081077	–	–	–
* Ceriporiopsis pseudoplacenta *	Miettinen 18997 (H)	KY948744	KY948902	KY948926	–	–
* Cerrena unicolor *	FD-299	KP135304	KP135209	KP134874	KP134968	–
* Climacodon sanguineus *	BR5020180728797	KX810931	KX810932	–	–	KX810934
* Climacodon septentrionalis *	AFTOL-767	AY854082	AY684165	AY864872	AY780941	AY885151
*Crustodontiachrysocreas* I	HHB-6333-Sp	KP135358	KP135263	KP134861	KP134908	–
*Crustodontiachrysocreas* II	FBCC307	LN611114	LN611114	–	–	–
* Daedalea quercina *	FP-56429	KY948809	KY948883	KY948989	–	–
* Datronia mollis *	RLG6304sp	JN165002	JN164791	JN164818	JN164872	JN164901
*Donkiapulcherrima* I	GC 1707-11	**LC378994**	**LC379152**	**LC379157**	**LC387351**	**LC387371**
*Donkiapulcherrima* II	AH39127	–	–	–	KX810937	–
*Donkiapulcherrima* II	Gothenburg-2022	KX752591	KX752591	–	–	–
* Efibula americana *	FP-102165	KP135016	KP135256	KP134808	KP134916	–
* Emmia lacerata *	FP-55521-T	KP135024	KP135202	KP134805	KP134915	–
* Fomitopsis pinicola *	AFTOL-770	AY854083	AY684164	AY864874	AY786056	AY885152
* Gelatoporia subvermispora *	FD-354	KP135312	KP135212	KP134879	–	–
*Geliporusexilisporus* I	GC 1702-15	**LC378995**	**LC379153**	**LC379158**	**LC387352**	**LC387372**
*Geliporusexilisporus* II	Dai 2172	KU598211	KU598216	–	–	–
* Gloeoporus pannocinctus *	L-15726-Sp	KP135060	KP135214	KP134867	KP134973	–
* Grammothelopsis puiggarii *	RP 134	KP859299	KP859308	–	–	–
* Hapalopilus nidulans *	FD-512	KP135419	–	KP134809	–	–
* Hapalopilus nidulans *	Josef Vlasak JV0206/2 (JV)	–	KX752623	–	–	–
* Hapalopilus ochraceolateritius *	Miettinen-16992.1	KY948741	KY948891	KY948965	–	–
* Heterobasidion annosum *	AFTOL-ID 470	DQ206988	–	DQ667160	–	DQ028584
* Heterobasidion annosum *	DAOM-73191	–	AF287866	–	AY544206	–
* Hydnophanerochaete odontoidea *	Chen 1376	**LC363485**	–	–		
* Hydnophanerochaete odontoidea *	GC 1308-45	**LC363486**	**LC363492**	**LC363497**	**LC387353**	**LC387373**
* Hydnophanerochaete odontoidea *	GC 1607-20	**LC378996**	–	–	–	–
* Hydnophanerochaete odontoidea *	GC 1710-59	**LC378997**	–	–	–	–
* Hydnophanerochaete odontoidea *	WEI 15-309	**LC378998**	–	–	–	–
* Hydnophanerochaete odontoidea *	WEI 15-348	**LC378999**	–	–	–	–
* Hydnophanerochaete odontoidea *	Wu 0106-35	**LC379000**	**LC379154**	LC379159	**LC387354**	**LC387374**
*Hydnophanerochaeteodontoidea* (*Phanerochaetesubodontoidea*)	Wu 911206-38	**LC379001**	–	–	–	–
* Hydnophanerochaete odontoidea *	Wu 9310-29	**LC379002**	–	–	–	–
* Hydnophanerochaete odontoidea *	Wu 9310-8	MF399408	GQ470653	LC314328	**LC387355**	**LC387375**
*Hydnophanerochaeteodontoidea* (*Phanerochaetesubodontoidea*)	CWN00776	**LC363487**	GQ470663	**LC363498**	**LC387356**	**LC387376**
* Hydnophlebia chrysorhiza *	FD-282	KP135338	KP135217	KP134848	KP134897	–
*Hydnophlebiaomnivora* I	KKN-112-Sp	KP135334	KP135216	KP134846	–	–
*Hydnophlebiaomnivora* II	ME-497	KP135332	KP135218	KP134847	–	–
* Hydnopolyporus fimbriatus *	Meijer3729 (O)	JN649346	JN649346	–	JX109875	JX109904
* Hyphoderma mutatum *	HHB-15479-Sp	KP135296	KP135221	KP134870	KP134967	–
* Hyphoderma setigerum *	CHWC 1209-9	–	–	–	**LC387357**	LC270919
* Hyphoderma setigerum *	FD-312	KP135297	KP135222	KP134871	–	–
* Hyphodermella corrugata *	MA- 24238	FN600378	JN939586	–	–	–
* Hyphodermella poroides *	Dai 10848	KX008368	KX011853	–	–	–
* Hyphodermella rosae *	FP-150552	KP134978	KP135223	KP134823	KP134939	–
* Irpex lacteus *	DO 421/951208 (O)	–	–	–	JX109882	JX109911
* Irpex lacteus *	FD-9	KP135026	KP135224	KP134806	–	–
* Leptoporus mollis *	TJV–93–174T	KY948795	EU402510	KY948957	–	–
*Lilaceophlebialivida* I	FBCC937	LN611122	LN611122	–	–	–
*Lilaceophlebialivida* II	FP-135046-sp	KY948758	KY948850	KY948917	–	–
* Lopharia cinerascens *	FP-105043-sp	JN165019	JN164813	JN164840	JN164874	–
* Luteoporia albomarginata *	GC 1702-1	**LC379003**	**LC379155**	**LC379160**	**LC387358**	**LC387377**
* Meruliopsis taxicola *	SK 0075 (GB)	JX109847	JX109847	–	JX109873	JX109901
* Merulius tremellosus *	ES2008-2 (GB)	JX109859	–	–	–	JX109916
* Merulius tremellosus *	FD-323	–	KP135231	KP134856	KP134900	–
* Mycoacia fuscoatra *	HHB-10782-Sp	KP135365	KP135265	KP134857	KP134910	–
* Mycoacia fuscoatra *	KHL 13275 (GB)	–	–	–	–	JX109908
* Mycoacia nothofagi *	HHB-4273-Sp	KP135369	KP135266	KP134858	KP134911	–
* Obba rivulosa *	FP-135416-Sp	KP135309	KP135208	KP134878	KP134962	–
* Odontoefibula orientalis *	GC 1604-130	**LC363489**	**LC363494**	**LC363500**	**LC387359**	**LC387378**
* Odontoefibula orientalis *	GC 1703-76	**LC379004**	**LC379156**	**LC379161**	**LC387360**	**LC387379**
* Odontoefibula orientalis *	Wu 0805-59	**LC363488**	**LC363493**	**LC363499**	**LC387361**	**LC387380**
* Odontoefibula orientalis *	Wu 0910-57	**LC363490**	**LC363495**	**LC363501**	**LC387362**	**LC387381**
* Odoria alborubescens *	BP106943	MG097864	MG097867	MG213724	MG213723	–
* Oxychaete cervinogilvus *	Schigel-5216	KX752596	KX752596	KX752626	–	–
* Phaeophlebiopsis caribbeana *	HHB-6990	KP135415	KP135243	KP134810	KP134931	–
* Phaeophlebiopsis peniophoroides *	FP-150577	KP135417	KP135273	KP134813	KP134933	–
* Phanerina mellea *	WEI 17-224	**LC387333**	**LC387340**	**LC387345**	**LC387363**	**LC387382**
* Phanerochaete arizonica *	RLG-10248-Sp	KP135170	KP135239	KP134830	KP134949	–
* Phanerochaete chrysosporium *	HHB-6251-Sp	KP135094	KP135246	KP134842	KP134954	–
* Phanerochaete ericina *	HHB-2288	KP135167	KP135247	KP134834	KP134950	–
* Phanerochaete exilis *	HHB-6988	KP135001	KP135236	KP134799	KP134918	–
* Phanerochaete laevis *	HHB-15519-Sp	KP135149	KP135249	KP134836	KP134952	–
* Phanerochaete livescens *	Wu 0711-81	**LC387334**	MF110289	**LC387346**	**LC387364**	LC270920
* Phanerochaete magnoliae *	HHB-9829-Sp	KP135089	KP135237	KP134838	KP134955	–
* Phanerochaete pseudosanguinea *	FD-244	KP135098	KP135251	KP134827	KP134942	–
* Phanerochaete rhodella *	FD-18	KP135187	KP135258	KP134832	KP134948	–
*Phanerochaete* sp.	HHB-11463	KP134994	KP135235	KP134797	KP134892	–
* Phanerochaete taiwaniana *	Wu 0112-13	MF399412	GQ470665	LC314332	**LC387365**	**LC387383**
* Phebia acerina *	FD-301	KP135378	KP135260	KP134862	–	–
*Phlebiaacanthocystis* I	GC 1703-30	**LC387338**	**LC387343**	–	**LC387366**	**LC387384**
*Phlebiaacanthocystis* II	FP150571	KY948767	KY948844	KY948914	–	–
* Phlebia albida *	GB-1833	KY948748	KY948889	KY948960	–	–
* Phlebia brevispora *	FBCC1463	LN611135	LN611135	–	–	–
* Phlebia centrifuga *	HHB-9239-Sp	KP135380	KP135262	KP134844	KP134974	–
* Phlebia coccineofulva *	HHB-11466-sp	KY948766	KY948851	KY948915	–	–
* Phlebia deflectens *	FCUG 1568	AF141619	AF141619	–	–	–
* Phlebia firma *	Edman K268	EU118654	EU118654	–	–	JX109890
* Phlebia floridensis *	HHB-9905-Sp	KP135383	KP135264	KP134863	KP134899	–
* Phlebia hydnoidea *	HHB-1993-sp	KY948778	KY948853	KY948921	–	–
* Phlebia lilascens *	FCUG 1801	AF141621	AF141621	–	–	–
* Phlebia ochraceofulva *	FBCC295	LN611116	LN611116	–	–	–
* Phlebia radiata *	AFTOL-484	AY854087	AF287885	AY864881	AY218502	AY885156
* Phlebia setulosa *	HHB-6891-Sp	KP135382	KP135267	KP134864	KP134901	–
*Phlebia* sp.	FD-427	KP135342	–	KP134849	–	–
*Phlebia* sp.	GC 1703-31	**LC387339**	**LC387344**	**LC387347**	**LC387367**	**LC387385**
*Phlebia* sp.	GC 1708-118	**LC387337**	**LC387342**	**LC387349**	**LC387368**	**LC387386**
*Phlebia* sp.	GC 1710-83	**LC387336**	**LC387341**	**LC387350**	**LC387369**	**LC387387**
*Phlebia* sp.	HHB-17984	KP135359	KP135261	KP134860	KP134907	–
*Phlebia* sp.	HHB-18295	KP135405	KP135269	KP134814	KP134938	–
*Phlebiasubochracea* I	HHB-8715-sp	KY948770	KY948846	KY948913	–	–
*Phlebiasubochracea* II	HHB-8494-sp	KY948768	KY948845	KY948912	–	–
* Phlebia subserialis *	FCUG 1434	AF141631	AF141631	–	–	–
* Phlebia uda *	FP-101544-Sp	KP135361	KP135232	KP134859	KP134909	–
* Phlebia unica *	KHL 11786 (GB)	EU118657	EU118657	–	JX109861	JX109889
* Phlebiopsis crassa *	KKN-86-Sp	KP135394	KP135215	KP134820	KP134928	–
* Phlebiopsis gigantea *	FP-70857-Sp	KP135390	KP135272	KP134821	KP134930	–
* Phlebiopsis ravenelii *	FP-110129-Sp	KP135362	KP135274	KP134850	KP134898	–
* Phlebiporia bubalina *	Dai 13168	KC782526	KC782528	–	–	–
* Pirex concentricus *	OSC-41587	KP134984	KP135275	KP134843	KP134940	–
* Rhizochaete filamentosa *	HHB-3169-Sp	KP135410	KP135278	KP134818	KP134935	–
* Rhizochaete radicata *	FD-123	KP135407	KP135279	KP134816	KP134937	–
* Rhizochaete rubescens *	Wu 0910-45	**LC387335**	MF110294	**LC387348**	**LC387370**	LC270925
* Riopa metamorphosa *	Viacheslav Spirin 2395 (H)	KX752601	KX752601	KX752628	–	–
* Sarcodontia crocea *	OMC-1488	KY948798	KY948903	KY948928	–	–
*Scopuloidesrimosa* I	HHB-7042-Sp	KP135350	KP135282	KP134853	KP134903	–
*Scopuloidesrimosa* II	RLG-5104	KP135351	KP135283	KP134852	KP134904	–
* Skeletocutis nivea *	ES2008-1 (GB)	JX109858	JX109858	–	JX109886	JX109915
* Steccherinum ochraceum *	KHL 11902 (GB)	JQ031130	JQ031130	–	JX109865	JX109893
* Stereum hirsutum *	AFTOL-ID 492	AY854063	–	AY864885	AY218520	AY885159
* Stereum hirsutum *	FPL-8805	–	AF393078	–	–	–
* Terana caerulea *	FP-104073	KP134980	KP135276	KP134865	KP134960	–
* Trametes versicolor *	FP-135156-sp	JN164919	JN164809	JN164825	JN164850	DQ028603
* Trametopsis cervina *	TJV–93–216T	JN165020	JN164796	JN164839	JN164877	JN164882
* Tyromyces chioneus *	FD-4	KP135311	KP135291	KP134891	KP134977	–

### *Phylogenetic analyses*

Two datasets were compiled for phylogenetic analyses: the ITS+nuc 28S+*rpb1*+*rpb2*+*tef1* dataset was analysed to confirm the generic placement of target species within the phlebioid clade of Polyporales. The ITS dataset was used to get better resolutions on species level within the *Hydnophanerochaete* clade of Meruliaceae. The selection of strains and species for the 5-marker dataset was based on Binder et al. (2013), Floudas and Hibbett (2015), Kuuskeri et al. (2015), Justo et al. (2017), Miettinen et al. (2016), Moreno et al. (2017), Papp and Dima (2017), Yuan et al. (2017) and Zhao et al. (2017). Alignment was done with MAFFT v. 7 using two strategies: Q-INS-I for ITS and FFT-NS-I for nuc 28S, *rpb1*, *rpb2* and *tef1* (Katoh and Standley 2013). The resulting alignments were manually adjusted in Mega 7 (Kumar et al. 2016). *Heterobasidionannosum* (Fr.) Bref. and *Stereumhirsutum* (Willd.) Pers., belonging to Russulales Kreisel ex P.M. Kirk, P.F. Cannon & J.C. David, were chosen as the outgroup in the 5-marker dataset. *Phlebiacoccineofulva* Schwein., belonging to Meruliaceae, was assigned as the outgroup in the ITS dataset. Optimised datasets were deposited at TreeBASE (submission ID 22932).

The Bayesian Inference (BI) method was carried out for both datasets using MrBayes v. 3.2.6 (Ronquist et al. 2012). The Maximum Likelihood (ML) method was carried out for the 5-marker dataset using RAxML BlackBox (Stamatakis 2014). For the BI analyses, jModeltest 2.1.10 (Darriba et al. 2012) was first used to estimate separate models for each of the markers in both datasets, based on Akaike information criterion (AIC). The Markov chain Monte Carlo (MCMC) search was run for ten million generations, with four chains and trees sampled every 100 generations. The first twenty-five percent of trees were discarded as burn-in while the remaining trees were used to construct the fifty percent majority-rule consensus phylogram with posterior probabilities (PP). For the ML analysis, the best-scoring tree with proportional values of bootstrap (BS) was computed under a GTRGAMMA model with one thousand bootstrap replicates, followed by a thorough ML search. Gaps were treated as missing data. Branches were regarded as having statistical support if values of PP and/or BS were equal to or over 0.9 and 70%, respectively. Both BI and ML analyses were performed at the CIPRES Science Gateway (Miller et al. 2010; http://www.phylo.org/). Phylograms were visualised and edited in TreeGraph 2 (Stöver and Müller 2010) and Adobe Illustrator (Adobe Systems, Inc).

## Phylogeny results

**Figure 1.**
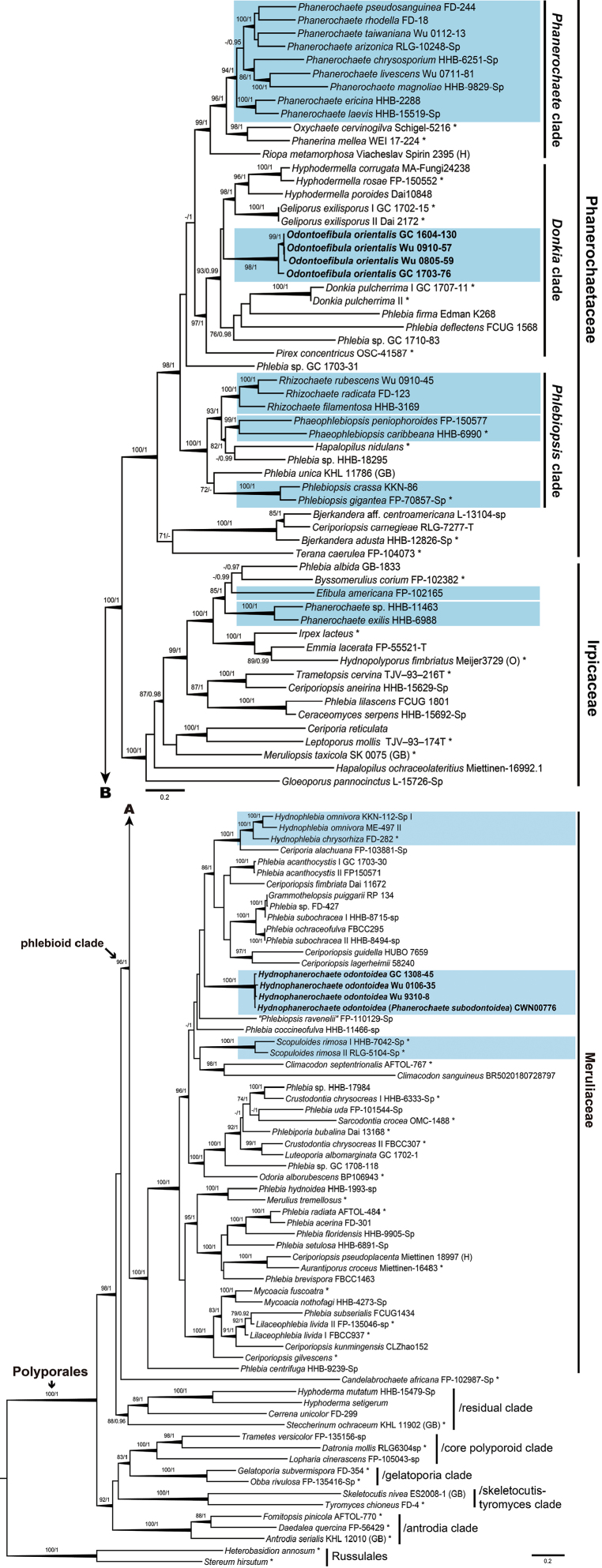
Phylogenetic tree inferred from Maximum Likelihood analysis of the combined ITS, nuc 28S, *rpb1*, *rpb2* and *tef1* sequences of taxa in Polyporales. Nodes are labelled with Maximum Likelihood bootstrap proportional values (BS) ≥ 70% and Bayesian Posterior Probabilities (PP) ≥ 0.9. Thickened branches obtained supports by both BS ≥ 80% and PP ≥ 0.95. The taxa studied in this study are shown in bold. The pale blue boxes indicate lineages of phanerochaetoid within the phlebioid clade. Asterisks (*) represent for strains of generic type species. Scale bars = substitutions per site.

The final ITS+nuc 28S+*rpb1*+*rpb2*+*tef1* dataset consisted of 126 sequences and 7253 characters (of which 43.7% were parsimony-informative) including gaps and the ITS dataset comprised 12 sequences and 887 characters (of which 7.7% were parsimony-informative) including gaps. In the BI analyses, since the GTR+G+I model was selected as the best model of nucleotide substitution for each of the five markers in the 5-marker dataset, it was used for the entire alignment with five partitions. The HKY+I+G model was selected as the best model of nucleotide substitution for the ITS dataset. The fifty percent majority-rule consensus phylogram with PP support values was reconstructed after the average standard deviation of split frequencies fell below 0.001. The best-scoring ML tree with BS support values was built. Phylogenetic trees of the 5-marker dataset, inferred from BI and ML algorithms, shared similar topologies and thus only the ML tree was shown (Fig. 1).

In the 5-marker analyses (Fig. 1), six main clades with high statistic supports (BS = 96–100%, PP = 1) could be recognised in the ingroup: the antrodia clade, the core polyporoid clade, the gelatoporia clade, the phlebioid clade, a residual clade and the skeletocutis-tyromyces clade. The phlebioid clade, which is the focus of this study, included three main subclades recognised as three families (BS = 100%, PP = 1): Irpicaceae, Meruliaceae and Phanerochaetaceae. *Hydnophanerochaeteodontoidea* formed a well-supported monophyletic lineage (BS = 100%, PP = 1) within Meruliaceae and was found to be closely related to a lineage consisting of strains of *Ceriporiaalachuana* (Murrill) Hallenb, *Ceriporiopsis* spp., *Grammothelopsispuiggarii* (Speg.) Rajchenb. & J.E. Wright, *Hynophlebia* spp. and *Phlebia* spp. (BS = 86%, PP = 1). Sequences of *Odontoefibulaorientalis* grouped together and formed a well-supported monophyletic lineage (BS = 98%, PP = 1) within the *Donkia* clade of Phanerochaetaceae (BS = 97%, PP = 1) and were most closely related to a lineage made up of strains of *Geliporusexilisporus* (Y.C. Dai & Niemelä) Yuan Yuan, Jia J. Chen & S.H. He and *Hyphodermella* spp. (BS = 98%, PP = 1).

**Figure 2.**
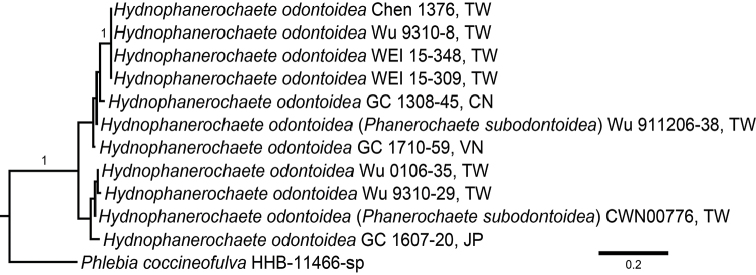
The majority-rule consensus phylograms of the Bayesian Inference analysis of the ITS sequences of *Hydnophanerochaeteodontoidea*. Nodes are labelled with Bayesian Posterior Probabilities ≥ 0.9. Scale bars = substitutions per site.

The tree inferred from the ITS dataset (Fig. 2) showed that sequences of holotype (*CWN00776*) and paratype (*Wu 911206-38*) of *Phanerochaetesubodontoidea* were clustered with sequences of *P.odontoidea* within a monophyletic lineage (PP = 1).

### Taxonomy

#### Hydnophanerochaete

Taxon classificationFungiORDOFAMILIA

Sheng H. Wu & C.C. Chen gen. nov.

MB824077

##### Type species.

*Hydnophanerochaeteodontoidea* (≡ *Phanerochaeteodontoidea*).

##### Etymology.

From hydnoid + *Phanerochaete*, referring to the hydnoid hymenial surface and a close affinity to *Phanerochaete*.

##### Description.

Basidiocarps effused, adnate, ceraceous. Hymenial surface at first buff, with age turning ochraceous to pale brown, slightly tuberculate to grandinioid when young, becoming odontioid to hydnoid with age, without colour changes in KOH. Aculei conical to cylindrical, ca. 1–4 per mm, up to 700 μm long.

Hyphal system essentially monomitic; generative hyphae simple-septate. Subiculum fairly uniform, composed of a basal layer, with compact texture; generative hyphae somewhat horizontal, colourless, thick-walled; quasi-binding hyphae present near substratum, colourless. Hymenial layer thickening. Trama of aculei of compact texture; generative hyphae somewhat vertical, colourless, thick-walled. Cystidia lacking, but projecting hyphal ends in the hymenium may be present. Basidia clavate, 4-sterigmate. Basidiospores ellipsoid to cylindrical, smooth, thin-walled, inamyloid, non-dextrinoid, acyanophilous.

##### Remarks.

*Hydnophanerochaete* is morphologically similar to the genus *Hydnophlebia* (Telleria et al. 2017). Both genera have resupinate basidiocarps with odontioid to hydnoid hymenial surface, a monomitic hyphal system, ordinarily simple-septate hyphae and similar basidiospore shape. However, we note three distinguishing differences. First, *Hydnophlebia* has membranaceous basidiocarps usually with rhizomorphic margin, while *Hydnophanerochaete* has ceraceous basidiocarps with fairly determinate margin. Second, occasional single or multiple clamp connections are present in subicular or aculei hyphae of *Hydnophlebia*, whereas they are lacking in hyphae of *Hydnophanerochaete*. Third, *Hydnophlebia* occasionally bears tubular to ventricose leptocystidia, which are lacking in *Hydnophanerochaete*.

Little morphological differences exist between *Hydnophanerochaete* and *Odontoefibula*: both genera have monomitic hyphal system with simple-septate hyphae and are lacking cystidia. However, *Hydnophanerochaete* is distinguished from *Odontoefibula* by its basidiocarps without colour change in KOH; additionally, its subiculum is compact, not dense.

*Phanerodontia* Hjortstam & Ryvarden, a recently proposed genus typified by *P.dentata* Hjortstam & Ryvarden (Hjortstam and Ryvarden 2010), is also morphologically similar to *Hydnophanerochaete*. However, the latter has a compact subiculum and quasi-binding hyphae near the substratum. *Phanerodontia* accommodates four species [*P.chrysosporium* (Burds.) Hjortstam & Ryvarden, *P.dentata*, *P.irpicoides* (Hjortstam) Hjortstam & Ryvarden and *P.magnoliae* (Berk. & M.A. Curtis) Hjortstam & Ryvarden], all of them possessing long leptocystidia (Hjortstam and Ryvarden 2010), whereas this structure is lacking in *Hydnophanerochaete*. Moreover, phylogenetically, strains of two species (*P.chrysosporium* and *P.magnoliae*) were recovered in Phanerochaetaceae which is only distantly related to *Hydnophanerochaete* (Fig. 1). However, the generic type has not been sequenced so far.

#### Hydnophanerochaete odontoidea

Taxon classificationFungiORDOFAMILIA

(Sheng H. Wu) Sheng H. Wu & C.C. Chen comb. nov.

MB824078

Figs 3a and 4

##### Basionym.

*Phanerochaeteodontoidea* Sheng H. Wu, Botanical Bulletin of the Academia Sinica 41: 169, 2000.

##### Synonym.

*Phanerochaetesubodontoidea* Sheng H. Wu, Botanical Bulletin of the Academia Sinica 41: 172, 2000.

##### Holotype.

TAIWAN. Ilan: Fushan Botanical Garden, 24°46’N, 121°35’E, 600 m alt., on fallen branch of angiosperm, leg. S.H. Wu et al., 7 Aug 1991, *Wu 910807-11* (TNM F14816).

##### Description.

Basidiocarps annual, effused, adnate, ceraceous, somewhat brittle, 50–200 μm thick in section (aculei excluded). Hymenial surface initially buff, with age turning ochraceous to pale brown, no colour changes in KOH, tuberculate to grandinioid when young, becoming odontioid to hydnoid with age, extensively cracked; margin paler to whitish, fairly determinate. Aculei conical to cylindrical, usually separate, with obtuse to acute apex, 1–4 per mm, up to 100–700 × 100–250 μm.

Hyphal system basically monomitic, some specimens with quasi-binding hyphae near substratum; generative hyphae simple-septate. Subiculum fairly uniform, composed of a basal layer of compact texture; generative hyphae mainly horizontal, colourless, 4–6 μm diam., with 0.8–1 µm thick walls; quasi-binding hyphae sometimes present near substratum, colourless, 1–3 µm diam. Hymenial layer thickening, with compact texture, generative hyphae somewhat vertical, colourless, 3–6 μm diam., slightly thick-walled. Trama of aculei of compact texture; generative hyphae mainly vertical, other features similar to those in subiculum; crystal masses present near apex. Cystidia lacking, but projecting hyphal ends in the hymenium may be present. Basidia clavate, 14–18 × 4.5–5.5 μm, 4-sterigmate. Basidiospores narrowly ellipsoid to cylindrical, adaxially slightly concave, smooth, thin-walled, homogeneous, inamyloid, non-dextrinoid, acyanophilous, 6–8.1 × 2.5–3.3 μm (Table 2). See also Wu (2000) for descriptions and illustrations.

**Figure 3.**
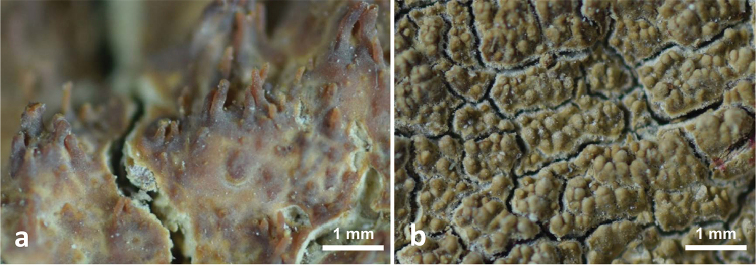
Basidiocarp surfaces **a***Hydnophanerochaeteodontoidea* (holotype of *Phanerochaetesubodontoidea*, *CWN 00776*) **b***Odontoefibulaorientalis* (holotype, *Wu 0910-57*). Scale bar: 1 mm.

**Figure 4.**
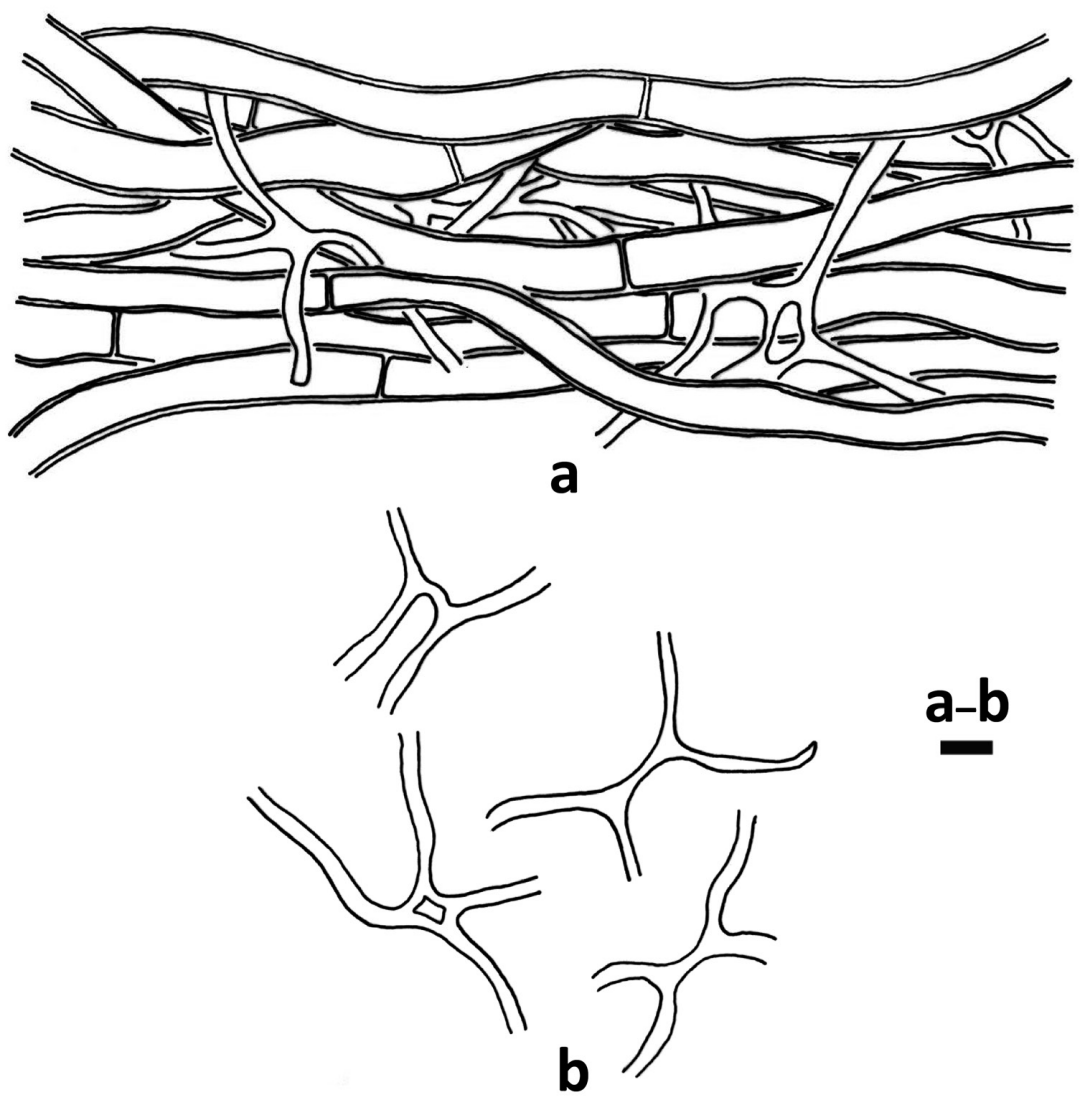
*Hydnophanerochaeteodontoidea* (holotype of *Phanerochaetesubodontoidea*, *CWN 00776*) **a** Part of the vertical section of subiculum near substratum **b** Quasi-binding hyphae. Scale bar: 5 μm (**a–b)**.

**Table 2.** Aculei and basidiospore measurements of basidiocarps.

Species	Specimens	Aculei (per mm)	Range (μm)	L (μm)	W (μm)	Q	n
* Hydnophanerochaete odontoidea *	Chen 1376	1–3	(6–) 6.3–7.3 (–7.5) ´ (2.5–) 2.8–3.3 (–3.5)	6.8	3	2.2	30
	CWN 00776 ^‡, |^	1–3	(6–) 6.8–8 (–8.5) ´ (2.5–) 2.7–3.2 (–3.5)	7.4	2.9	2.5	30
	GC 1308-45 ^|^	2–3	(6.5–) 6.7–7.6 (–8) ´ (2.8–) 2.8–3.3 (–3.8)	7.2	3.1	2.3	30
	GC 1607-20	2–3	(7–) 7.4–9 (–10) ´ (2.8–) 2.9–3.5 (–4)	8.2	3.2	2.6	30
	WEI 15-309	2–3	(6–) 6.1–7 (–7.5) ´ (2.5–) 2.7–3 (–3.3)	6.5	2.9	2.3	30
	WEI 15-348	2–3	6–6.9 (–7.5) ´ (2.5–) 2.8–3.3 (–3.5)	6.5	3	2.1	30
	Wu 0106-35 ^|^	2–3	(6–) 6.4–7.8 (–8) ´ (2.5–) 2.8–3.1 (–3.3)	7.1	2.9	2.4	30
	Wu 910807-11 ^†^	3–4	(6–) 6.1–7 (–8) ´ (2.5–) 2.5–2.9 (–3.3)	6.5	2.7	2.5	30
	Wu 911206-38 ^‡^	2–3	(6–) 6.3–7.7 (–8) ´ (2.8–) 2.9–3.2 (–3.5)	7	3	2.3	30
	Wu 9310-8 ^†, |^	2–4	(6–) 6.5–8 (–8.5) ´ (2.5–) 2.8–3.2 (–3.5)	7.2	3	2.4	30
	Wu 9310-29	2–4	(6–) 6.9–8.1 (–9) ´ (2.5–) 2.7–3.3 (–3.7)	7.4	3	2.5	30
* Odontoefibula orientalis *	GC 1604-130 ^|^	4–5	(5–) 5.4–6.6 (–7) ´ (2.5–) 2.8–3.3 (–3.6)	6	3.1	1.96	30
	GC 1703-76 ^|^	4–5	(5.5–) 5.8–7.4 (–8) ´ (3–) 3.2–3.9 (–4)	6.6	3.5	1.85	30
	Wu 0805-59 ^|^	3–5	(5–) 5.1–6.2 (–7) ´ (2.5–) 2.9–3.4 (–3.6)	5.6	3.2	1.79	30
	Wu 0807-53	3–6	(5–) 5.4–6.4 (–7) ´ (3–) 3.1–3.7 (–4)	5.9	3.4	1.71	30
	Wu 0910-57 ^§, |^	3–6	(5–) 5.4–6.1 (–6.5) ´ (2.8–) 2.9–3.4 (–3.6)	5.7	3.2	1.81	30

^†^ Holotype and paratype of *Phanerochaeteodontoidea*.

^‡^ Holotype and paratype of *P.subodontoidea*.

^§^ Holotype of *Odontoefibulaorientalis*.

^|^ Used in phylogenetic analyses of the 5-marker dataset.

##### Habitat.

On fallen branches of angiosperms or gymnosperms.

##### Distribution.

Hitherto known from subtropical to temperate regions of China (Yunnan), Japan, Taiwan and Vietnam.

##### Additional specimens examined.

CHINA. Yunnan: Diqing Tibetan Autonomous Prefecture, Deqin County, Xiayubeng Village, Shenhu Trail, 3500 m alt., on fallen branch of gymnosperm, leg. C.C. Chen, 14 Aug 2013, *GC 1308-45* (TNM F27660). JAPAN. Honshu: Nagano Prefecture, Nagano City, Myoko-Togakushi Renzan National Park, 36°45’35”N, 138°04’20”E, 1235 m alt., on branch of *Quercus* sp., leg. C.C. Chen & C. L. Chen, 29 July 2016, *GC 1607-20* (TNM F30785). TAIWAN. Chiayi: Yushan National Park, Nanhsi Forest Road, 23°28’N, 120°54’E, 1850 m alt., on fallen branch of angiosperm, leg. S.H. Wu & S.Z. Chen, 13 Oct 1993, *Wu 9310-8* (paratype of *P.odontoidea*, TNM F14824); *Wu 9310-29* (TNM F14826); 1800 m alt., on fallen branch of angiosperm, leg. S.H. Wu & S.Z. Chen, 13 Jun 1996, *Wu 9606-55* (TNM F5085). Ilan: Fushan Botanical Garden, 24°46’N, 121°35’E, 650 m alt., on fallen branch of angiosperm, leg. S.H. Wu et al., 28 Jun 2002, *Wu 0106-35* (TNM F13460). Nantou: Tungpu Township, Leleku, 1450 m alt., on fallen rotten wood, leg. W.N. Chou, 13 Apr 1994, *CWN 00776* (holotype of *P.subodontoidea*, TNM F14836). Kaohsiung: Maolin District, Tona Nursery, 22°54’N, 120°44’E, 850 m alt., on fallen branch of angiosperm, leg. S.Z. Chen, 31 Mar 2005, *Chen 1376* (TNM F18764). New Taipei: Chinshan District, Yangmingshan National Park, Yulu Historical Trail, 25°10’N, 121°35’E, 516 m alt., on fallen branch of angiosperm, leg. C.C. Chen, C.L. Wei, W.C. Chen & S. Li, 26 Aug 2015, *WEI 15-309* (TNM F29370); *WEI 15-348* (TNM F29384). Taichung: Chiapaotai, 850 m alt., on fallen branch of angiosperm, leg. S.H. Wu, 6 Dec 1991, *Wu 911206-38* (paratype of *P.subodontoidea*, TNM F14818). VIETNAM. Lam Dong: Bi Doup Nui Ba National Park, 12°10’45”N, 108°40’48”E, 1447 m alt., on fallen branch of angiosperm, leg. C.C. Chen, 15 Oct 2017, *GC 1710-59* (TNM F31365).

##### Remarks.

*Phanerochaetesubodontoidea* morphologically resembles *Phanerochaeteodontoidea*, whereas they were distinguished merely based on the width of basidiospores [*P.odontoidea*: 2.6–3 µm vs. *P.subodontoidea*: 3–3.7 µm, Wu (2000)]. However, after carefully measuring the basidiospore size of available specimens of these two species, we found basidiospore ranges are highly overlapping (Table 2). Additionally, the ITS sequences of the holotype of *P.subodontoidea* (*CWN 00776*) is almost identical to the ITS sequences of the paratype of *P.odontoidea* (*Wu 9310-8*). We failed to obtain sequences from the holotype of *P.odontoidea* (*Wu 910807-11*), but *Wu 9310-8* was confirmed as conspecific with the holotype by morphological comparison. Thus, based on morphological and molecular evidence (Fig. 2), *P.subodontoidea* is treated as a synonym of *P.odontoidea*. A paratype specimen named *P.odontoidea* (*Wu 9311-46*) probably belongs to the genus *Flavodon* Ryvarden based on preliminary BLAST results of nuc 28S sequences. However, this specimen was not included in this study.

#### Odontoefibula

Taxon classificationFungiORDOFAMILIA

C.C. Chen & Sheng H. Wu gen. nov.

MB824075

##### Type species.

*Odontoefibulaorientalis*.

##### Etymology.

From *odonto* (= tooth-like) + *efibula* (= without clamp connection), referring to the odontioid hymenial surface and simple-septate hyphae of the genus.

##### Description.

Basidiocarps annual, resupinate, effused, adnate, membranaceous to ceraceous. Hymenial surface at first honey yellow, becoming ochraceous to pale brown with age, turning dark reddish in KOH, initially smooth to slightly tuberculate, becoming grandinioid to odontioid with age. Aculei conical to cylindrical, separate or fused, up to 0.3 mm long.

Hyphal system monomitic; hyphae normally simple-septate. Subiculum uniform, with dense texture; basal hyphae interwoven, somewhat horizontal or with irregular orientation, colourless, thin- to slightly thick-walled; subicular hyphae somewhat vertical, colourless, thin- to slightly thick-walled. Subhymenium not clearly differentiated from subiculum. Central trama of fairly dense texture; hyphae vertical, colourless, thin- to slightly thick-walled. Cystidia lacking, but projecting hyphal ends in the hymenium may be present. Basidia clavate to narrowly clavate, 4-sterigmate. Basidiospores ellipsoid, smooth, thin-walled, inamyloid, non-dextrinoid, acyanophilous.

##### Remarks.

*Phaneroites* Hjortstam & Ryvarden, a monotypic genus introduced to accommodate *P.subquercinus* (Henn.) Hjortstam & Ryvarden, resembles *Odontoefibula* in having odontioid hymenial surface and a monomitic hyphal system with ordinarily simple-septate hyphae. However, *Phaneroites* is distinguished from *Odontoefibula* by having thin-walled subicular hyphae, a few clamped septa on hyphae next to the substratum and subcapitate cystidia (Hjortstam and Ryvarden 2010). Moreover, basidiocarps of *Odontoefibula* turn dark reddish in KOH, while this reaction was not reported from *Phaneroites*.

#### Odontoefibula orientalis

Taxon classificationFungiORDOFAMILIA

C.C. Chen & Sheng H. Wu sp. nov.

MB824076

Figs 3b and 5

##### Holotype.

CHINA. Beijing: Xiangshan Park, 39°59’N, 116°11’E, 70 m alt., on fallen trunk of *Amygdalusdavidiana* (Carrière) de Vos ex Henry, leg. S.H. Wu, 14 Oct 2009, *Wu 0910-57* (TNM F23847).

##### Etymology.

From *orientalis* (= Eastern world), where the specimens were collected.

##### Description.

Basidiocarps annual, effused, adnate, membranaceous to subceraceous, somewhat brittle, 200–400 μm thick in section (aculei excluded). The hymenial surface at first honey yellow, darkening to ochraceous to pale brown with age, turning dark reddish in KOH, slightly tuberculate when young, becoming odontioid with age, extensively cracked; margin paler, thinning out, slightly filamentous. Aculei conical to cylindrical, usually fused at the base, with rounded to obtuse apex, 3–6 per mm, ca. 0.1–0.3 × 0.1–0.2 mm.

Hyphal system monomitic; hyphae simple-septate. Subiculum uniform, with dense texture, 200–300 μm thick; subicular hyphae somewhat vertical, colourless, 2.5–4 μm diam., 0.5–0.8 μm thick walls; hyphae near substratum interwoven, with irregular orientation, tortuous, colourless, irregularly swollen, 4–8 μm diam., 0.5–1 μm thick walls. Subhymenium not clearly differentiated from subiculum, with fairly dense texture, hyphae somewhat vertical, colourless, 3–4 μm diam., thin- to slightly thick-walled. Trama of aculei of dense texture; hyphae mainly vertical, other aspects similar to those in subiculum. Large crystal masses scattered throughout the section. Cystidia lacking, but projecting hyphal ends in the hymenium may be present. Basidia clavate to narrowly clavate, 25–40 × 6–7 μm, 4-sterigmate, often with small oily drops. Basidiospores ellipsoid, adaxially slightly concave, smooth, thin-walled, sometimes with small oily drops, inamyloid, non-dextrinoid, acyanophilous, 5.1–6.6 × 2.8–3.4 μm (Table 2).

**Figure 5.**
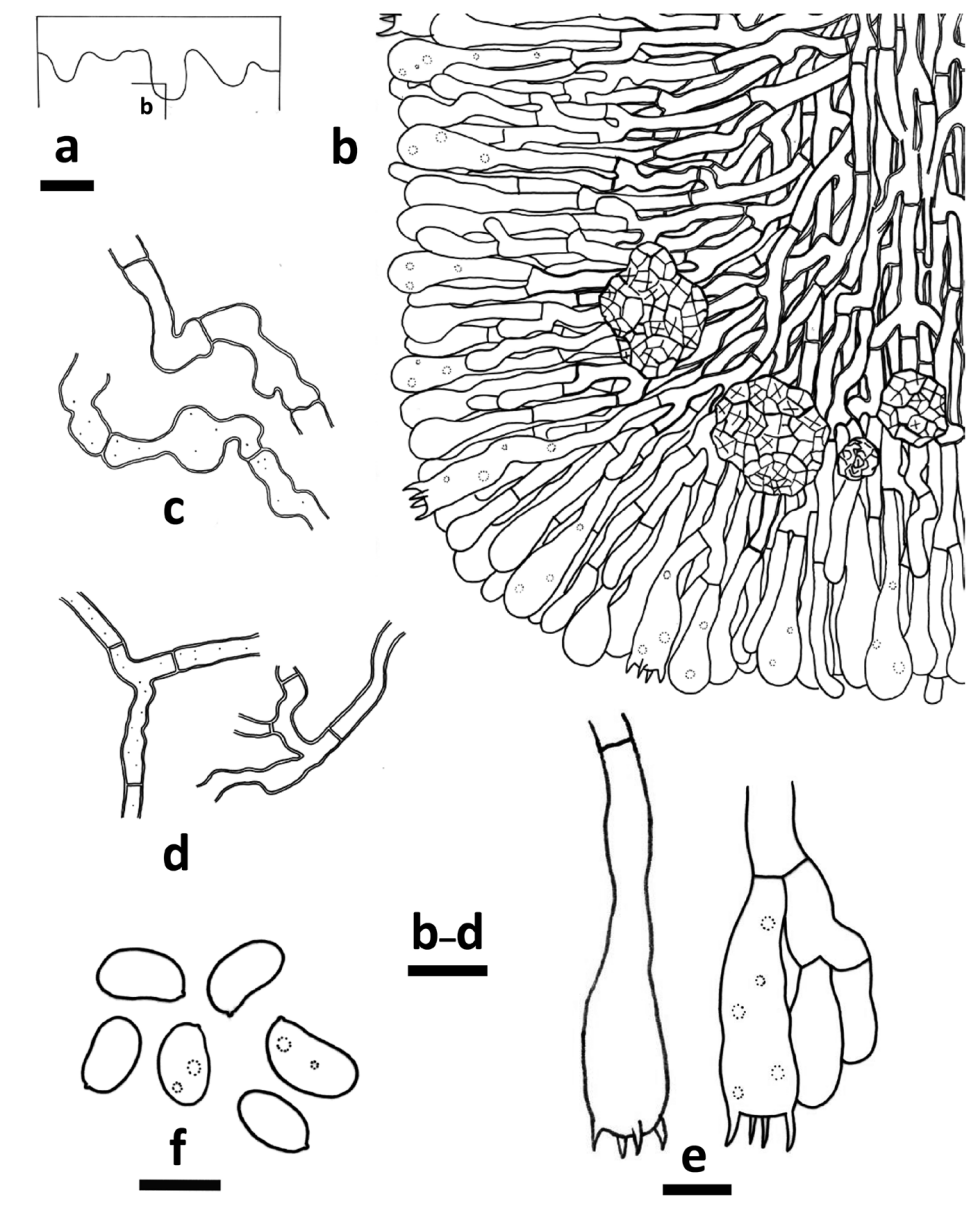
*Odontoefibulaorientalis* (holotype, *Wu 0910-57*) **a** Profile of basidiocarp section **b** Part of the vertical section of trama **c** Basal hyphae **d** Subicular hyphae **e** Basidia **f** Basidiospores. Scale bars: 200 μm (**a**); 10 μm (**c–d)**; 5 μm (**e–f)**.

##### Habitat.

On fallen trunk of angiosperm (e.g. *Amygdalus*).

##### Distribution.

Hitherto known from China (Beijing), Japan and Taiwan.

##### Additional specimens examined (paratypes).

JAPAN. Honshu: Ibaraki Prefecture, Joso City, Mt. Ju-ichimen-yama, along Kinu-gawa River, on branch of *Prunus* sp., leg. S.H. Wu, 12 July 2008, *Wu 0807-53* (TNM F22091). TAIWAN. Pingtung: Laiyi Township, Pengjishan Trail, 22°30’52”N, 120°38’07”E, 248 m alt., on fallen trunk of angiosperm, leg. C.C. Chen, 25 Mar 2017, *GC 1703-76* (TNM F31460). Taichung: Hoping District, between 27–27.5 km of Dasyueshan Forestry Road, Yuanzueishan Trail, 1800 m alt., on fallen rotten trunk of angiosperm, leg. S.H. Wu, S.Z. Chen & Y.T. Wang, 22 May 2008, *Wu 0805-59* (TNM F22495). Hualien: Sioulin Township, Taroko National Park, Lushui Hiking Trail, 24°10’51”N, 121°30’10”E, 578 m alt., on fallen trunk of angiosperm, leg. C.C. Chen, 24 Apr 2016, *GC 1604-130* (TNM F31364).

## Discussion

Our 5-marker phylogenetic analyses (Fig. 1) provided an updated taxonomic framework for evaluating generic placements of the target taxa of the phlebioid clade. The tree topologies are consistent with previous results (Wu et al. 2010; Floudas and Hibbett 2015; Justo et al. 2017; Papp and Dima 2017). Within the phlebioid clade, we recovered two monophyletic lineages of phanerochaetoid fungi (Fig. 1), which supports the status of the two genera erected here: *Hydnophanerochaete*, typified by *P.odontoidea*, is accommodated in Meruliaceae; *Odontoefibula*, typified by *O.orientalis*, is placed in *Donkia* clade of Phanerochaetaceae.

Phylogenetically, *Hydnophanerochaete* and *Odontoefibula* are independent from the nine lineages of phanerochaetoid fungi recognised by Floudas and Hibbett (2015) within the phlebioid clade: *Efibula*, *Hydnophlebia*, *Phaeophlebiopsis*, “*Phanerochaete*” *allantospora* Burds. & Gilb., *Phanerochaete* s.l., *Phanerochaete* s.s., *Phlebiopsis*, *Rhizochaete* and *Scopuloides*. *P.allantospora* was not sampled in this study; it was placed in Irpicaceae, according to the study of Justo et al. (2017). Additionally, “*Phanerochaete*” *ginnsii* Sheng H. Wu represents another lineage of phanerochaetoid fungi that was not analysed in this study, nor in the study of Floudas and Hibbett (2015). This species was shown to be closely related to *Phlebiacentrifuga* P. Karst (Wu et al. 2010).

The 5-marker phylogenetic analyses (Fig. 1) suggest a close relationship amongst *Hydnophanerochaeteodontoidea* and the following taxa, which all have a monomitic hyphal system with simple-septate hyphae: *Hydnophlebia*, *Ceriporiaalachuana*, *Climacodonseptentrionalis* (Fr.) P. Karst. and *Scopuloidesrimosa* (Cooke) Jülich. Like *Hydnophanerochaete*, *Hydnophlebia* and *Scopuloides* have an odontioid to hydnoid hymenial surface. However, *Hydnophlebia* differs by its membraneous basidiocarps with rhizomophic margin, occasional clamped subicular hyphae and the presence of tubular to ventricose leptocystidia (Telleria et al. 2017). *Scopuloides* differs by thick-walled encrusted cystidia and rather short, clavate basidia (Wu 1990). *C.alachuana* resembles *H.odontoidea* in lacking cystidia, but has a poroid hymenial surface (Ryvarden and Gilbertson 1993). *C.septentrionalis* has a hydnoid hymenial surface, but is clearly distinguished by its pileate basidiocarps and thick-walled encrusted cystidia (Maas Geesteranus 1971).

Quasi-binding hyphae, one of the diagnostic characters of *H.odontoidea* (Fig. 4), were first introduced by Wu (1990) to refer to narrow and much branched subicular hyphae with thin- to thick walls, found near the substrate. Wu (2000) omitted describing and illustrating the quasi-binding hyphae of *P.odontoidea* and *P.subodontoidea*. Quasi-binding hyphae have been reported from many species of diverse genera: *Amethiciumleoninum* (Burds. & Nakasone) Sheng H. Wu, *Crustodontiachrysocreas* (Berk. & M.A. Curtis) Hjortstam & Ryvarden, *Phlebiporiabubalina* Jia J. Chen, B.K. Cui & Y.C. Dai, *Phanerochaeteericina* (Bourdot) J. Erikss. & Ryvarden, *Pseudolagarobasidiumcalcareum* (Cooke & Massee) Sheng H. Wu and *Radulodonamericanus* Ryvarden (Wu 1990; Stalpers 1998; Chen and Cui 2014). In other words, this feature has a polyphyletic origin and does not seem to be very phylogenetically informative.

Within the *Donkia* clade (Fig. 1), systematic positions of two recently proposed taxa, *Geliporusexilisporus* and *Hyphodermellaporoides* Y.C. Dai & C.L. Zhao, are confirmed in this study. *Odontoefibula* shares some ubiquitous features with the genera *Donkia*, *Hyphodermella* J. Erikss. & Ryvarden and *Pirex* Hjortstam & Ryvarden, many of which have ochraceous basidiocarps with odontioid to hydnoid hymenial surfaces. However, to better illustrate the correspondence between molecular data and morphology, denser taxon sampling of this clade is necessary in the future.

## Supplementary Material

XML Treatment for Hydnophanerochaete

XML Treatment for Hydnophanerochaete odontoidea

XML Treatment for Odontoefibula

XML Treatment for Odontoefibula orientalis
